# Managing life during the pandemic: communication strategies, mental health, and the ultimate toll of the COVID-19

**DOI:** 10.3906/sag-2106-175

**Published:** 2021-12-17

**Authors:** Mehmet Hakan TÜRKÇAPAR, Yasemin KAHYA, Tuğba ÇAPAR-TAŞKESEN, Hatice IŞIK

**Affiliations:** 1 Department of Psychology, Faculty of Social Sciences and Humanities, Social Sciences University of Ankara, Ankara Turkey

**Keywords:** Coronavirus disease-19, health communication, mental health, posttraumatic growth, psychological intervention, protective factors

## Abstract

**Background/aim:**

The purpose of this review was to present the ultimate toll of the COVID-19 pandemic by focusing on the communication strategies and mental health.

**Materials and methods:**

We unsystematically reviewed the studies published between 2020 and 2021 from databases such as Google Scholar, Web of Science and ScienceDirect. Firstly, “new-normal” life challenges during the pandemic were discussed along with the public risk communication strategies. Later, mental health problems, posttraumatic growth, and protective factors were reviewed.

**Results:**

Literature highlighted that individuals mainly experience COVID-19 related fear, anxiety, stress, negative emotions and sleep problems. Furthermore, the rates of clinically significant depression, anxiety, obsessive compulsive disorder, and posttraumatic stress disorder suggest an increase. Specifically, COVID-19 stress syndrome, loneliness, and sleep problems were associated with mental health problems in the pandemic. However, some individuals seem to be resilient to the COVID-19 trauma and experience posttraumatic growth. Brief online intervention studies are promising for reducing the emotional toll of the COVID-19 as well as for making individuals more resilient.

**Conclusion:**

To conclude, the negative conditions of the pandemic seem to make some people, but not all, vulnerable to mental illness. In addition, framing the public warnings in an optimal emotional tone seems to be more effective to comply with the precautions.

## 1. Introduction

Pandemic is generally defined as an epidemic that occurs worldwide and affects a large number of people at the same time [1]. The coronavirus outbreak (The COVID-19), which was first detected in Wuhan, China in December 2019 World Health Organization (2020). Pneumonia of Unknown Cause – China [online]. Website https://www.who.int/csr/don/05-january-2020-pneumonia-of-unkown-cause-china/en/ [accessed 26 May 2021]., has continued to be a pandemic that affects the whole world World Health Organization (2021). WHO Coronavirus (COVID-19) Dashboard [online]. Website https://covid19.who.int/ [accessed 12 Jun 2021].. According to the World Health Organization, 174.918.667 people have been infected with this disease until now and more than 3,7 million have died because of coronavirus globally, as of 12 June 2021^2^. In addition, studies have shown that in some cases, people who have recovered from coronavirus may experience deterioration in their physical health and psychological well-being [2]. Considering the fear, stress, and anxiety created by the COVID-19 pandemic [3,4], as well as the global and local precautions to protect public health (e.g., lockdown, wearing a mask, etc.), people’s mental health has also been negatively affected by this stressful period. Therefore, in these days, it is important to examine the consequences of the pandemic and the psychological experiences of people in order to suggest local and global precautions immediately. In this article, firstly, the difficulties experienced by individuals during the pandemic will be discussed and then the risk communication strategies will be explained. Finally, mental health problems associated with the COVID-19 outbreak, the COVID-19 related posttraumatic growth, and protective factors will be mentioned with the possible intervention methods. 

## 2. The ultimate toll of the COVID-19

The high visibility of deaths and infected people during the COVID-19, combined with intolerance to uncertainty, has increased people’s anxiety and stress levels [5,6]. However, those are not the only reasons affecting negatively mental health. With the global and local precautions taken in this phase, people experience many different extraordinary changes and faced different obstacles in daily life. 

In addition to witnessing deaths and infected cases every day, people around the world have suffered psychological, social and economic consequences. Since the virus is transmitted very quickly from person to person, WHO and national governments have frequently emphasized the importance of keeping physical distance from other people World Health Organization (2020). Coronavirus Disease (COVID-19) Advice For The Public [online]. Website https://www.who.int/emergencies/diseases/novel-coronavirus-2019/advice-for-public [accessed 29 May 2021].. For this reason, some countries have applied nationwide lockdowns with different time periods. Even in regions where those lockdowns were not declared legally, people began to go out less to protect themselves and their acquaintances from the disease [7]. The fact that people are in lockdown, whether legally or voluntarily, has caused psychological and economic consequences [8]. Many of the people had to work from home in this process which might result in inequalities in the labor market by favoring older individuals, males, and individuals with higher education [9]. Besides, studies found that the gender gap in satisfaction with job and productivity increased in the working from home process. That is, women stated lower satisfaction and productivity in their job compared to men [10]. The reason for this gap is compatible with expectations of the society from different genders. That is, while men can spare time for their work as before at home settings, women are expected to fulfill responsibilities related to housework and children as well as continue to do their job. 

Working from home, economic difficulties, gender gap between couples and spending too much time at home with family members are new and unusual situations emerging due to the COVID-19 pandemic. Therefore, the relationships between the family members may be negatively affected by those new situations. For example, studies indicated that domestic violence has increased during pandemic, indicating a higher risk for children and their mothers End Violence Against Children (2020). Protecting Children During COVID-19 [online]. Website https://www.end-violence.org/protecting-children-during-covid-19-outbreak [accessed 29 May 2021]. [11,12]. Experiencing difficulties related to the COVID-19 might cause dyadic hostility and withdrawal while decreasing supportive response between couples. This might influence couples’ relationship quality negatively by laying more stress on preexisting problems (e.g., extended family issues, social status) and individual vulnerabilities (e.g., insecure attachment style) [13]. 

Education from kindergarten to university has gone through major transformations during the COVID-19 pandemic. Some countries closed all education institutions and have been resuming education online as a precaution to contain the spread of the infection UNESCO (2021). Education: From Disruption To Recovery [online]. Website https://en.unesco.org/covid19/educationresponse#durationschoolclosures [accessed 30 May 2021].. Those education-related closures have affected more than 90% of the student population worldwide United Nations (2020). Policy Brief: Education During COVID-19 and Beyond [online]. Website https://www.un.org/development/desa/dspd/wp-content/uploads/sites/22/2020/08/sg_policy_brief_covid-19_and_education_august_2020.pdf [accessed 29 May 2021].. Difficulty in accessing technological tools (e.g., tablets, internet) during remote education, the lack of face-to-face interaction with the instructor and absence of educational and social stimulus have been making students more vulnerable to the negative consequences of pandemic and increasing the risk of drop-out rates at schools OECD Policy Responses to Coronavirus (COVID-19) (2020). The Impact of COVID-19 on Student Equity and Inclusion: Supporting Vulnerable Students during School Closures and School Re-openings [online]. Website https://www.oecd.org/coronavirus/policy-responses/the-impact-of-covid-19-on-student-equity-and-inclusion-supporting-vulnerable-students-during-school-closures-and-school-re-openings-d593b5c8/ [accessed 06 Jun 2021]..

The healthcare sector is another area where employees are very tired both psychologically and physically World Health Organization (2020). Protecting Health Workers from COVID-19 [online]. Website https://www.who.int/westernpacific/news/feature-stories/detail/protecting-health-workers-from-covid-19 [accessed 04 Jun 2021].. According to the WHO’s statement in May 2021, it is estimated that more than 150,000 healthcare workers have been infected by the virus in this process [14]. Healthcare employees also have struggled with various psychological disorders such as depression, anxiety, and stress [15] with the fear of infecting their loved ones [16]. In addition, people with diseases such as cancer, diabetes, or hearing loss had difficulty in accessing health care [17–19], and some patients delayed their treatment due to the fear of the COVID-19 virus [20,21]. This fear has caused many patients to deliberately underestimate their symptoms. As a result, they were exposed to delayed diagnosis, treatment, higher risk of complications and eventually they died [22]. Even if they go to the hospital, diagnostic procedures and access to technical facilities may be delayed due to special precautions for the COVID-19 patients [23]. 

As a result, many precautions have been taken to prevent the spread of the COVID-19 infection all over the world, highlighting the importance of social distance, but, naturally, this has caused some negative economical and psychological consequences. Many people have to spend most of their time at home, either voluntarily or compulsorily, to comply with these measures. This situation combined with economic problems negatively affected physical and mental health of the individuals and resulted in social isolation. 

In this extraordinary period, the importance of obtaining accurate information about COVID-19 has become even more important in order to ensure that the society adheres to these measures. This drew attention to the communication strategies used by experts when sharing information about the COVID-19 disease to promote public health. 

## 3. The communication strategies during the COVID-19 pandemic

It is of great importance to ensure that the measures recommended by health authorities are complied with by the community to prevent the spread of the COVID-19 pandemic. How this information is communicated to the community is also crucial to provide compliance with the precautions. This communication type refers to “the exchange of real-time information, advice and opinions between experts and people facing threats to their health, economic or social well-being” World Health Organization (n.d). Risk Communication [online]. Website https://www.who.int/emergencies/risk-communications [accessed 05 Jun 2021].. Given the emotional, cognitive and behavioral dimensions of individuals’ responses in situations that threaten their health or safety [24,25], it seems that risk communication during the COVID-19 is not just about providing statistical and scientific information to the public. More than a one-way transfer of information, it is a two-way interaction in which experts and the public guide each other [26]. In other words, the behavior of the public may differ according to how the information is being provided by the experts. Authorities need to observe public reaction to risk communication processes to update their communication strategies in order to promote public health. Cultural and contextual differences should be considered when using these communication strategies [27]. Effective risk communication is especially important for developing countries as a preventative factor because these countries may find it difficult to overcome increased hospitalization and intensive health care [28]. Therefore, the implementation of the measures by the public in these countries by relying on the explanations of experts might reduce the spread of the COVID-19 and decrease the burden on the health sector. 

The dimension emphasized by the experts during risk communication is effective in the decision of the public whether to apply the warnings in their life. Realistic information such as statistical data and scientific research can be used to motivate change as well as to emphasize lively, tangible, and personal elements that touch the emotional aspects [29]. Studies have shown that emotional emphasis is more effective than providing realistic information when warning people about risky situations [30]. For example, higher fear from the virus predicted positive behavior change such as social distancing and promoted hand-washing in the COVID-19 pandemic [31]. Attention should be given to the intensity of the emotional framework of those COVID-19 warnings, otherwise it may increase the stress and anxiety in the public too much. It has been observed that people with high fear and anxiety during the COVID-19 engage in extreme behaviors such as hoarding toilet papers, medicines or face masks [32,33]. Thus, experts need to arouse *feelings* towards the risks on the one hand, and at the same time give a sense of trust to the public [34], which is difficult to balance in a crisis situation. In this case, it is necessary to use an appropriate emotional tone as well as realistic information so that the public can trust the experts and social policy makers and comply with the suggested precautions.

In addition to how the message is framed during the COVID-19, the characteristics of the audience must also be considered in this process. An important aspect of the ability to cope with potentially dangerous events is the desire for getting information about the threatening situation. Researchers divided people into two categories regarding how to approach danger related information. Some people, referred to as monitors, have a tendency to search out information and focus on threatening aspects of the condition, whereas blunters have a tendency to avoid information [35]. People with a tendency to seek information and to consider threats (monitors), and those who do not seek detailed information about risks (blunters), may react differently to risk communication strategies. A study showed that the monitors tended to perceive more risk than blunters when the emotional dimensions of risk were emphasized, but when realistic information was given, there was no difference between the risk perception of the two groups [30]. This could be speculated that emotionally weighted messages with realistic information would be more effective on a certain group of society during the COVID-19 pandemic. Personality traits are another factor that plays a role in the interpretation of experts’ warnings about the COVID-19. For example, one study found that people who score high on the agreeableness and conscientiousness dimension gave more attention to health messages related to the COVID-19 and approved of measures such as social distancing [36]. Considering that individuals pay more attention to persuasive messages in line with their own views [37], it can be expected that people will follow those that match their personality traits when evaluating health-related warnings during a crisis such as the COVID-19 pandemic. For this reason, it is necessary to consider individual differences while preparing these warnings and determining risk communication strategies. Considering the different personality traits, cognitive skills and emotional sensitivities in the society, the development of different risk communication methods instead of standard and monotonous messages would increase compliance with the recommended measures related to COVID-19.

One of the biggest challenges during the COVID-19 pandemic was trying to control rumors and misinformation about the disease, which increased the perception of uncertainty, pseudoscientific ideas, and conspiracy theories [38]. Those rumors and misinformation related to the causes, consequences and treatment of the disease spread very quickly among people, especially via social media [39]. For example, despite the lack of any valid evidence, many people around the world believed that the virus was spread by 5G technologies [40], or the aim of the vaccination is to insert a chip to the people. In addition, misinformation that some “miraculous” foods (such as garlic, minerals, drinkable silver) protect people from the COVID-19 virus was shared on many social media platform BBC-Reality Check (2020). Koronavirüs (Covid-19): Virüs ve Hastalık Hakkında Dikkate Almamanız Gereken Hurafeler [online]. Website https://www.bbc.com/turkce/haberler-dunya-51815676 [accessed 06 Jun 2021].. Those kinds of misinformation about the disease, treatment options, and vaccines might result in decreased health behaviors and increased inaccurate practices in the public. Thus, refutation of those misinformation and rumors is crucial to promote public health globally. In this way, experts provide an integrity image to the public and encourage protective attitudes towards the COVID-19 pandemic by increasing positive emotions and perceived efficacy [41]. To disprove rumors, experts must first have the latest research findings and accurate information. In order to convey this information to the public in an accurate and effective way, the cooperation between the mass media and health institutions is crucial [42]. In addition, the rapid detection of nonscientific online content from all social media platforms will facilitate their intervention.

## 4. Mental health problems

The COVID-19 outbreak and related outcomes such as fear and anxiety of getting infected, social isolation and other restrictive life-changing precautions have negatively affected the well-being of individuals. World Happiness Report [43] documented that there was an increase in different forms of *“negative affect”* (stress, worry, and sadness) in the global sample of countries. In the context of the COVID-19 outbreak and deterioration in well-being, mental health problems become one of the ultimate tolls of the pandemic. 

We can think of psychological responses to the COVID-19 outbreak on a spectrum ranging from indifference to extreme anxiety. The initial normal reactions to this abnormal life-changing situation may show itself as heightened stress, fear and anxiety, increased depressive symptoms and substance use, sleep problems, loss of control, feelings of hopelessness, helplessness and loneliness [44]. Data from the world indicated that the initial reaction to the expectations of lockdown was the increase in psychological distress and mental illness [45,46]. However, the trajectory of mental health effects has been changing as the course and restrictions of the COVID-19 outbreak change. Supportively, longitudinal data collected during the lockdown showed that there was a slow recovery in depression and anxiety symptoms a few weeks after the lockdown [47]. Thus, a moderate level of fear or anxiety may help individuals in coping with threats in the context of the COVID-19 [48], without risking mental health clinically. On the contrary, clinically significant mental health problems, that is the severe and persistent emotional responses, can emerge depending on the personal vulnerabilities and other circumstances surrounding the COVID-19 outbreak.

Depression, anxiety, stress, sleep problems, obsessive compulsive disorder (OCD)-related conditions, health anxiety, and posttraumatic stress disorder (PTSD) were commonly researched mental health problems during the current pandemic. In the following section, we first review the recent data on clinically significant levels of depression and anxiety, followed by data on OCD-related conditions and health anxiety. Finally, we will review available data on PTSD, which is a great risk for patients who recovered from intensive care, and also for health care workers during the COVID-19 outbreak. We presented systematic reviews and meta-analysis findings where possible. 

### 4.1. Depression, anxiety, and sleep problems

The meta-analysis of Bueno-Navitol et al. [49] indicated that the rates of clinically significant depression in the general population considerably increased during the outbreak, with even higher rates than other pandemics in history. The comprehensive meta-analysis of Cénat et al. [50], including data from 68 independent samples, demonstrated that women and men populations who were affected from the COVID-19 outbreak had higher rates of depression, anxiety, panic disorder and insomnia compared to rates before the outbreak. The global cross-sectional study of Varma et al. [51], including data from more than 60 countries, showed that even if the number of cases in each particular country varied, high rates of stress, state anxiety, depression, and poor sleep were consistent across the globe. In this global cross-sectional study, sleep quality and loneliness were important vulnerability factors to poorer mental health. The meta-analysis of Chang et al. [52] examined studies on depression and anxiety among college student samples from different countries. Depressive and anxiety symptom prevalence were the highest for American students; anxiety symptom prevalence was the lowest for Chinese students, and depression symptom prevalence was the lowest for Turkish students. These various prevalence rates among countries may be partially explained by the government precautions. Supportively, the metaanalysis of Lee et al. [53] reported that fast and strict government precautions to control the spread of the COVID-19 reduce mental health problems. This is meaningful in the sense that physical and psychological well-being cannot be separated. The reduced uncertainty and increased safety feelings via government precautions may facilitate mental health during the COVID-19. These metaanalyses findings generally express that individuals globally report higher rates of clinically significant levels of depression and anxiety, but the rates of depression and anxiety may show some differences across countries.

In terms of depression, anxiety and sleep problems, individuals who have survived after a severe COVID-19 infection, healthcare workers, and pregnant women need special interest. The meta-analysis of Liu et al. [54] documented that depression, anxiety and insomnia symptoms were highly prevalent in the COVID-19 patients, even higher than the general population affected by the outbreak. In this meta-analysis, insomnia symptoms and being a female come to the forefront. In an umbrella review of meta-analysis studies on healthcare workers by Sahebi et al. [55], the summary of findings highlighted the vulnerability of medical staff to depression and anxiety due to workload, burnout, and the fear of contracting the disease as well as stigmatization and isolation from the loved ones. The rapid review and meta-analysis by Thomfohr-Madsen et al. [56] documented that pregnant women experienced elevated levels of depression and anxiety during the COVID-19. Specifically, there was a trend in increase in anxiety levels of pregnant women as the COVID-19 process extends, probably because of chronic stress in pandemic times and ongoing uncertainty. Pregnant women in Europe and North America were more vulnerable to pregnancy anxiety than women in Asia [56]. 

Thus, the risk for clinically significant levels of depression and anxiety as well as sleep problems seem to be heightened in different segments of the society during the COVID-19. Currently, people deal with fear surrounding the disease, lockdown, fluctuations in the number of infected people and deaths. In these uncontrollable conditions, society feels a lack of control and deals with financial difficulties and uncertainty about the future, all of which may explain the increase in depression and anxiety rates during the new coronavirus outbreak.

### 4.2. OCD and health anxiety

The COVID-19 pandemic may be specifically threatening for individuals who have predisposition to OCD and health anxiety. Psychologically, the uncertainty and unpredictability of the disease and related conditions need to be tolerated along with behavioral precautions such as hand washing and using disinfectants frequently. The uncertainty about the disease and behavioral precautions during the outbreak may exacerbate pre-existing OCD and health anxiety or contribute to the development of these mental health problems. To a certain degree, healthy individuals feel fear and anxiety for getting infected and transmitting the infection to loved ones. However, in this context, individuals having predisposition to OCD and health anxiety may feel extreme stress because of the nature of their mental health problems [57,58].

Taylor et al. [59] developed the COVID-19 stress scale, measuring the pandemic related stress in different domains: (a) danger and contamination fears, (b) fears about economic consequences, (c) xenophobia, (d) compulsive checking and reassurance seeking, and (e) traumatic stress symptoms about the COVID-19. In the initial scale development study, OCD symptoms and health anxiety were associated with the COVID-19 related stress. More recent examinations also corroborated that OCD symptoms, health anxiety, and fear of the COVID-19 were all correlated [60].

Although reporting mixed results, following studies mostly supported first formulations of Taylor at al. [59]. The study by Acenowr and Coles [61] supported that high severity of OCD symptoms and OC-related intrusive thoughts resulted in greater severity in and distress on the COVID-19-related intrusive thoughts. The study by Fontenelle et al. [62] further showed that being female, the number of the COVID-19-related stressful events, and pre-existing fear of harm and symmetry symptoms before the outbreak predicted OCD symptoms during the COVID-19, except fear of contamination and washing. Also, the rates of clinically significant OCD, hoarding disorder (HD), and skin picking disorder (SPD) escalated as well as already existing OCD, HD, SPD, and hair pulling worsened after the pandemic.

Another study by Khosravani et al. [63] documented the increase in all OCD dimensions, namely contamination, responsibility for harm, unacceptable thoughts, and symmetry. On the contrary, Wheaton, Ward et al. [64] found that contamination and responsibility for harm symptoms worsened more than taboo thoughts or symmetry symptoms after the COVID-19. However, authors also reported a considerable variability in the responses to the COVID-19 among individuals with self-identified OCD. In this regard, the majority of individuals with self-identified OCD reported a slight worsening while fewer of them reported substantial worsening or no change, reflecting a heterogeneity in reactions to the COVID-19. Mask wearing, hand washing, and physical distancing that decrease the risk of the COVID-19 disease infection may explain the development and exacerbation of contamination obsessions and phobias, and OCD symptom severity during the COVID-19 outbreak [65].

Thus, the current literature implies that the preexisting OCD and health anxiety features, especially danger and contamination threat, may heighten the COVID-19 stress syndrome, that is, the fear of spread of disease. The COVID-19 disease may also result in an increase in the rates of OCD related phenomenon, meaning that people may develop new OCD symptoms. Preexisting OCD symptoms may indicate a slight worsening, but studies are inconsistent whether all or particular OCD dimensions tend to get worsened since individual reactions have varied according to data.

### 4.3. Posttraumatic stress disorder (PTSD)

The novel coronavirus, SARS-CoV-2, leads to the COVID-19 disease that is highly infectious, has caused deaths, and poses future death threats. Therefore, the COVID-19 disease can be considered as a traumatic event, probably more for coronavirus survivors and healthcare workers. A metaanalysis supported that three of the ten coronavirus survivors, two of the ten healthcare workers, and one of the ten individuals in the general population reported to have a PTSD diagnosis or PTSD related symptoms [66]. This metaanalysis, inclusive studies on SARS, MERS and the COVID-19, provided data on the increase in the prevalence rates of PTSD during outbreaks. The increase in PTSD-related conditions during outbreaks has also been repeated by research specific to the COVID-19 [50]. During the recent coronavirus pandemic, females, people who had poor sleep quality and/or who live in widely affected regions, people who got infected and/or people who work with the COVID-19 patients were more vulnerable to PTSD [67]. Thus, PTSD may be one of the mental health tolls caused by the COVID-19 outbreak, which may increase substance use problems and suicide risk.

### 4.4. Who are vulnerable to mental health problems when facing the COVID-19?

#### 4.4.1. Age, gender, and pre-existing mental health problems

In general, younger age compared to middle or older age and being female in the Middle East and the West constituted vulnerability factors to mental health problems across global studies [51,68–72]. These results might be explained by the increased feelings of loneliness in the context of the COVID-19, social isolation, and financial difficulties among poorer young people [51] and demanding cultural gender roles for women in the Middle East and some regions of the West. 

Clinical and empirical studies during the COVID-19 commonly support that individuals with preexisting mental health problems were more prone to the COVID-19 negative mental health effects [57,62,73]. Due to the pre-existing cognitive vulnerabilities of overestimation of threat, inflated sense of responsibility, intolerance of uncertainty, over-importance of thoughts and their control, the COVID-19 was more likely to result in negative reactions to the pandemic, exacerbation or acquisition of anxiety-related disorders [57]. In the high-disease risk context of COVID-19, preexisting OCD-related problems worsened, with patterns of more disability, affective problems, and reduced quality of life [62]. Having a pre-existing mental health problem before the pandemic showed a trajectory of more severe depression and anxiety symptoms during the pandemic [73]. As well as the COVID-19 disease fear, anxiety, and isolation, the limited access to mental health services may explain the exacerbation of mental health problems among individuals with preexisting mental health problems. Thus, those individuals are more vulnerable to extreme negative pandemic reactions, exacerbation of symptoms, and relapse in COVID-19 context. 

#### 4.4.2. Personality and intolerance to uncertainty

To the authors knowledge, studies examining the effect of *personality* on the clinically significant levels of mental health problems during the COVID-19 have been limited. Available research addressed the role of *neuroticism, psychoticism and detachment *on the mental health problems associated with the COVID-19 outbreak. In the earlier study of Mazza et al. [74], negative affectivity and detachment were associated with internalizing symptoms, assessed during the COVID-19 lockdown. In the study of Nikčević et al. [75] neuroticism was found to be related to the COVID-19 anxiety, health anxiety, and generalized anxiety and depression symptoms. One caveat in the mentioned study is that the COVID-19 anxiety was itself associated with anxiety and depression symptoms, independently from neurotic personality features. In the study of Somma et al. [76], neuroticism and psychoticism, assessed at the beginning of the lockdown, significantly predicted internalizing symptoms at the end of the lockdown. Although some personality features seem to be related to mental health problems, they seem not to fully explain the variance in those problems. Future research should examine personality factors as the course of the COVID-19 has been changing.

*Intolerance to uncertainty* has been commonly assessed as a vulnerability factor to mental health problems during the COVID-19. This dispositional incapacity is a triggered aversive response by the absence of sufficient information and this aversive response is sustained by the lack of endurance to the perception of uncertainty [77]. Different studies reported that intolerance to uncertainty was associated with the COVID-19 stress syndrome [59], reduced general well-being [78] and internalizing symptoms [6]. Wheaton, Messner et al. [60] further indicated that intolerance to uncertainty during the COVID-19 explained the associations between OCD symptoms, health anxiety, and fear of the disease. Along with intolerance to uncertainty, other cognitive vulnerabilities such as overestimation of threat and perceptions of low ability to cope were related to mental ill-being during a real COVID-19 disease threat [61]. Furthermore, Han et al. [79] showed the association between risk perception and emotional distress and Schmidt et al. [80] between cognitive anxiety sensitivity (racing thoughts may be interpreted as losing one’s mind) and pandemic related stress. Thus, maladaptive cognitive-emotional styles seem to be particularly important for mental health in the COVID-19.

## 5. Posttraumatic growth in the COVID-19

Given that the COVID-19 was considered as a traumatic event, studies conducted by mental health researchers have focused on this mass trauma’s effects on people. In addition to the emergence of negative individual consequences after traumatic events, another possibility is posttraumatic development or growth. The literature supports that the traumatic life events not only result in psychological problems such as PTSD, depression and anxiety but also give an opportunity to people to develop themselves in a positive way by improving the quality of their interpersonal relationships. As in the previous section, the negative impacts of the COVID-19 have been clarified, the following part focuses on the positive effects defined mostly as “posttraumatic growth (PTG)”. PTG is a positive psychological transformation that has occurred as a result of struggling with traumatic life events [81]. After a traumatic event that shutter previous perception of the world such as having a feeling of being secure and safe, people try to adapt to the “new normal” by improving deeper relationships, having much personal strength, looking for new opportunities, strengthening a sense of spirituality and appreciating life [81]. During the COVID-19, the PTG was measured in different time periods. Also, the PTG data was collected from different samples like students [82], the COVID-19 patients, the general population [83] and healthcare workers [84] which is also divided into subgroups based on their closeness to the COVID-19 patients. According to the literature, in a study conducted in October 2020 with students who graduated from high school, about 13% of the students had reported PTG besides depression and anxiety [82]. The other research, examined PTG among the COVID-19 confirmed patients via using interview method, revealed that confirmed patients had reported PTG. Mainly three growth areas appeared: 1) reevaluating priorities such as reviewing their aims, values and being grateful for living, 2) developing deeper relationships in their social circle, 3) improvements in personal chance such as gaining maturity and having more awareness of the significance of health [85]. Further research on the general population (40% were healthcare workers) in April 2020 showed that the whole participants reported moderate to low PTG and about 28% of the general population exhibited PTSD symptoms. Having found all participants showed PTG, further analysis was conducted to compare the healthcare workers and nonhealthcare workers in terms of their PTG scores in the mentioned study. In the personal strength dimension, a component of PTG and defined as feeling stronger compared to the past, healthcare workers were found to experience significantly more growth compared to non-healthcare workers. Another comprehensive research investigated the PTG among nurses [84]. Parallel with the previous studies, about 40% of participants had reported PTG experience during the COVID-19 pandemic. At the same time, a high percentage of the sample (39.3%) reported trauma and the responders also stated a moderate level of emotional exhaustion. In addition, nurses who worked at hospitals designed to care for patients with the COVID-19, who worked in a critical unit or who gave care for patients with the COVID-19 had expressed significantly higher levels of total PTG compared to the other nurses who did not work in these units. 

In the light of the aforementioned studies, healthcare workers are more inclined to experience PTG besides more mental health problems since they have to cope with both the COVID-19 related stressors such as threat of being infected and also the fear of spreading this infection to their loved ones [83]. Furthermore, healthcare workers who have worked in isolated hospitals designed for patients with the COVID-19 or cared for these patients encounter vicarious trauma [86] since some of their patients received traumatic treatments and some of them died [83]. In addition to these adversities, health personnel exhibited positive psychological change; however, there is a curvilinear relationship in PTG. Therefore, both health organizations and the government should take more advanced precautions for healthcare workers to mitigate physical and psychological impacts of the COVID-19 pandemic. 

## 6. Protective factors and interventions 

The ongoing COVID-19 outbreak has caused detrimental effects on not only physical but also psychological health. A substantial body of study highlighted that the pandemic and its restrictions were related to a greater level of depression, anxiety, stress, posttraumatic stress disorder, sleep disturbances, burnout, suicidal ideation, loneliness. On the other hand, a large-scale study underscored that not everyone was evenly influenced by the COVID-19 outbreak [87]. According to the results of this research, being women, single, younger, having more children, having received a lower level of education, residing in a rural area were risk factors during the pandemic. They were affected by more severely COVID-19 related stressors and reported greater levels of stress. In addition to the risk factors, the protective factors were also identified and listed. Resilience, active coping skills, exercise, social support, self-efficacy, stable income are accounted as a protective shield against the effects of the COVID-19 [88-91].

Having determined the risk and protective elements for the COVID-19, various interventions were introduced and/or implemented. Some interventions targeted specifically one aspect of mental consequences of the COVID-19 such as loneliness; however, most of them focused on the more general mental health problems. For instance, LOVE was a brief intervention designed to improve social connectedness to overcome loneliness reported mostly during the COVID-19 [92]. It consists of four stages: list everyone in one’s life, organize them according to their availability/helpfulness, verify the significant others and engage to these significant others via self-disclosure (the first letters of these four stages are abbreviated for the name of the program: LOVE). Similarly, cognitive behavior therapy for insomnia was tailored to the COVID-19 to mitigate sleep disturbances and ameliorate sleep quality [93]. In addition to these interventions specifically focused on some elements of outbreak, mindfulness, yoga, music intervention [94]and cognitive behavior therapy [95] were applied to overall mental health problems such as anxiety and depression. 

Furthermore, the other interventions highlight the importance of enhancing protective factors since they can buffer the adverse psychological distress. Several seminal studies underscored the term of resilience defined as the ability to remain healthy in the presence of stress and adversity [96]. The literature has suggested that higher level of resilience was associated with better mental health [97], higher wellbeing [88], and greater preventative effects on developing mental health disorders [98]. Thus, research conducted during the outbreak undertakes to show how to increase resilience skills among the population [88,99,100]. They gave some recommendations in terms of personal (e.g., mindfulness, implementing active coping skills), organizational (e.g., supplying protective equipment and relaxation activities), and environmental levels (e.g., room settings). The interventions also pointed out emotional regulation strategies [101], improving self-compassion such as gratitude [102,103] and stress related coping mechanisms [95]. 

Because of the restrictions during the pandemic, most of the psychological interventions have taken place on the internet so researchers also shift their attention towards the online-therapy processes. Due to its nature, the pandemic has affected everyone around the world. However, the source of psychological support is very scarce worldwide. In this regard, online brief therapies, even only one-session interventions, gained importance in the psychology field. Researchers who have studied the subject of online therapies found that one session intervention programs [103] as well as the other brief therapies/interventions [104] alleviated the psychological impact of the pandemic. These preliminary results are very promising.

## 7. Conclusion

Since December 2019, unfortunately, the COVID-19 infection has spread throughout the world and has had detrimental consequences on mental health with different pathways [43]. As a first pathway to mental health problems, individuals experience health-related anxieties, that is, the fear and anxiety of the COVID-19 disease, which result in debilitating physical symptoms, hospitalization, threats of losing loved ones after contagion. The other pathway may be financial worries and stress such as losing jobs and reduction in payments, to which disadvantaged socio-economic classes of the society may be more vulnerable. The third pathway may be the changes in the living conditions at home such as meeting the demands of working and parenting at the same time. The fourth pathway is related to mental health effects of lockdown due to the loss or otherwise limited-fulfilling activities. This extraordinary period made it more crucial to get accurate information in a public mental health sensitive way about the COVID-19 globally.

Uncertainty and unpredictability, personal distress, fear and anxiety and health-related worries have increased during the COVID-19 outbreak. These circumstances constitute a vulnerability context for mental health problems. The summary of implications from the present narrative review is presented in the Figure. Immense empirical research on mental health outcomes of the COVID-19 have proliferated. Research should continue to examine mental health outcomes with regard to longitudinal and persistent effects. Majority of research used various self-report measures to determine clinical risk and to to arrive at a conclusion without diagnostic assessments and also, some of the studies that were included in systematic reviews and meta-analyses lacked adequate quality. Review of the findings should be understood with these limitations. Mental health research based on rigorous methods should be one of the future aims in the COVID-19 research. However, there is enough research evidence that national and international community health agendas should put mental health issues at the center. Clinically significant mental health problems in the context of the COVID-19 need intervention and professional psychological support.

**Figure:**
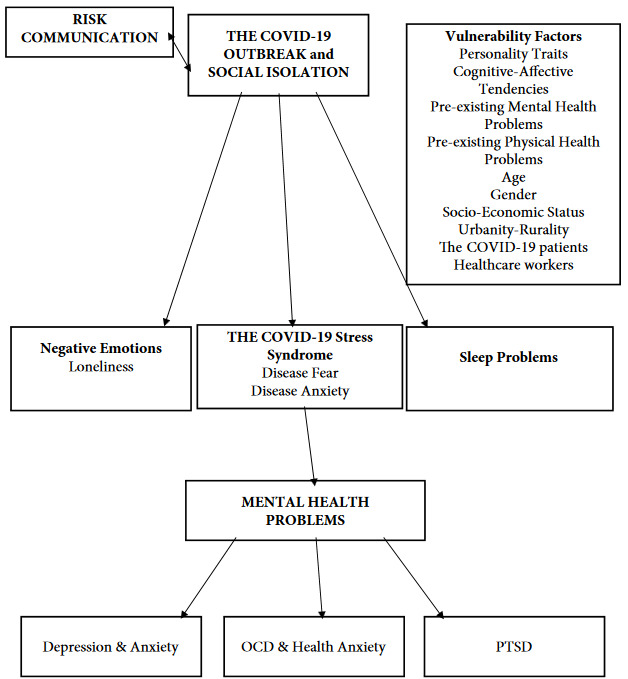
The mental health toll of the COVID-19 outbreak.

Beside detrimental effects of traumatic events, positive mental health outcomes could be observed such as improved relationships. This phenomenon is known as PTG and it can also be accounted as a protective factor. Thus, PTG can be included in brief interventions programs. The COVID-19 pandemic has affected many people in different ways. Hence, tailored interventions should have been applied to people after determining the risk and protective factors in detail. In the psychology literature, resilience has been accepted as a skill and can be developed via interventions [105]. Therefore, building and maintaining this skill in the general population is highly recommended during these days. Furthermore, online brief or one session therapies and interventions have been gaining popularity in these mass trauma days. The research investigating single-session interventions on mental health supported various psychological effects. For instance, a study examining the psychological effects of online single-session interventions (such as psycho-education and anxiety management) showed that participants who received the intervention had a decrease in anxiety symptoms and negative affect, while there was no difference in positive affect and well-being [104]. The other single-session intervention study, which was designed to use behavioral activation, gratitude and cognitive restructuring techniques, revealed an increase in the well-being of the participants [103]. In the light of the mentioned studies, there was improvement in different areas in the participants according to the targeted method in intervention programs. In future studies, brief interventions may focus on COVID 19’s negative consequences in the first part of the session/s and positive effects in the second part of session/s or vice versa. 

## Informed consent

This is a review article. Therefore, the study protocol did not receive institutional review board approval. Since no investigation was conducted with humans, no informed consent was required.
